# ABCE1 Acts as a Positive Regulator of Exogenous RNA Decay

**DOI:** 10.3390/v12020174

**Published:** 2020-02-04

**Authors:** Takuto Nogimori, Koichi Ogami, Yuka Oishi, Ryoya Goda, Nao Hosoda, Yoshiaki Kitamura, Yukio Kitade, Shin-ichi Hoshino

**Affiliations:** 1 Department of Biological Chemistry, Graduate School of Pharmaceutical Sciences, Nagoya City University, Nagoya 467-8603, Japan; tnogimori@nibiohn.go.jp (T.N.); koichi_ogami@phar.nagoya-cu.ac.jp (K.O.); yuka.oishi0720@gmail.com (Y.O.); ryoya.goda@gmail.com (R.G.); hosoda@phar.nagoya-cu.ac.jp (N.H.); 2 Laboratory of Immunosenescence, National Institutes of Biomedical Innovation, Health and Nutrition, Ibaraki-City, Osaka 567-0085, Japan; 3 Department of Biomolecular Science, Graduate School of Engineering, Gifu University, 1-1 Yanagido, Gifu 501-1193, Japan; kitamura@gifu-u.ac.jp (Y.K.); ykkitade@aitech.ac.jp (Y.K.); 4 Department of Applied Chemistry, Faculty of Engineering, Aichi Institute of Technology, 1247 Yachigusa, Yakusa-cho, Toyota, Aichi 470-0392, Japan

**Keywords:** ABCE1, oligoadenylate synthetase, Ribonuclease L, exogenous RNA, RNA quality control

## Abstract

The 2′-5′-oligoadenylate synthetase (OAS)/RNase L system protects hosts against pathogenic viruses through cleavage of the exogenous single-stranded RNA. In this system, an evolutionally conserved RNA quality control factor Dom34 (known as Pelota (Pelo) in higher eukaryotes) forms a surveillance complex with RNase L to recognize and eliminate the exogenous RNA in a manner dependent on translation. Here, we newly identified that ATP-binding cassette sub-family E member 1 (ABCE1), which is also known as RNase L inhibitor (RLI), is involved in the regulation of exogenous RNA decay. ABCE1 directly binds to form a complex with RNase L and accelerates RNase L dimer formation in the absence of 2′-5′ oligoadenylates (2-5A). Depletion of ABCE1 represses 2-5A-induced RNase L activation and stabilizes exogenous RNA to a level comparable to that seen in RNase L depletion. The increased half-life of the RNA by the single depletion of either protein is not significantly affected by the double depletion of both proteins, suggesting that RNase L and ABCE1 act together to eliminate exogenous RNA. Our results indicate that ABCE1 functions as a positive regulator of exogenous RNA decay rather than an inhibitor of RNase L.

## 1. Introduction

The innate immune system is made of host defenses against infection that can be activated immediately by recognizing pathogen-associated molecular patterns (PAMPs) or danger-associated molecular patterns (DAMPs). In higher vertebrates, the innate immune system depends on interferon (IFN)-signaling pathways. Among the first discovered IFN-induced antiviral defense mechanisms, the canonical 2′-5′-oligoadenylate synthetase (OAS)/RNase L system is an RNA cleavage pathway that responds to viral dsRNAs as PAMPs [1,2]. In response to the dsRNAs, OAS produces a unique oligonucleotide 2′-5′ oligoadenylates (2–5A), which acts as a second messenger to trigger dimerization and activation of latent RNase L. RNase L endonuclease cleaves viral single-stranded RNA and limits viral replication [3,4]. Recently, an RNA quality control factor Dom34/Pelota was identified as a restriction factor for a positive-sense single stranded RNA virus [5]. Pelota functions in selective targeting of exogenous viral RNA: Pelota directly binds RNase L to form a surveillance complex to recognize and eliminate the exogenous RNA in a manner dependent on translation [5]. 

On the other hand, it has been reported that the activity of RNase L is modulated by ATP-binding cassette sub-family E member 1 (ABCE1) also known as RNase L inhibitor (RLI). ABCE1/RLI was identified from screening of an expression library by the ability to bind 2-5A. ABCE1/RLI was found to antagonize the binding of 2-5A with RNase L and partially inhibit RNase L [6]. Nevertheless, it seems that ABCE1 does not readily bind 2-5A and does not compete for it [6]. Furthermore, ABCE1 does not degrade 2-5A [6]. In addition, previous reports have shown that ABCE1 influences the viral replication process [6,7,8], but little has been reported on whether ABCE1 affects the stability of viral RNAs. 

Besides the inhibition of RNase L, ABCE1 has diverse functions: HIV capsid assembly, ribosome biogenesis, translation initiation and tissue homeostasis [9,10,11,12,13]. Notably, two papers by Khoshnevis et al. and Pisarev et al. reported that ABCE1 has a novel important role in translation termination and ribosome recycling by dissociating ribosomes into large and small subunits [14,15]. Since then, the function of ABCE1 has been extensively investigated as a ribosome recycling factor [14,16,17,18,19]. ABCE1 interacts with eukaryotic releasing factor (eRF)1 or Pelota to dissociate ribosomes either after translation termination of normal mRNAs or after recognition of stalled ribosomes on aberrant mRNAs, respectively [14,16,18]. Interestingly, RNase L interacts with the translation factors: Pelota and eRF3 (a cofactor of eRF1) [5,20]. In addition, we have recently shown that RNase L degrades exogenous RNAs in a manner dependent on translation [5]. The fact that both ABCE1 and RNase L are closely related to translation prompted us to consider the idea that ABCE1 might be a cofactor rather than an inhibitor of RNase L. 

Here, we show that ABCE1 directly interacts with RNase L and Pelota to function in the decay of exogenous RNA. Our results indicate that ABCE1 acts as a positive regulator of exogenous RNA decay rather than an inhibitor of RNase L. 

## 2. Materials and Methods

### 2.1. Cell Culture and Transfection

HeLa cells (CCL-2, ATCC) were grown in Dulbecco’s modified Eagle’s medium (Nissui Pharmaceutical, Tokyo, Japan) supplemented with 5% fetal bovine serum at 37 °C and 5% CO_2_. Transfection of siRNA and in vitro transcribed RNA was performed using Lipofectamine RNAiMAX (Invitrogen Life Technologies, Carlsbad, CA, USA) according to the manufacturer’s instruction. Transfection of 2-5A and plasmid DNA was performed using Neon^TM^ Transfection System (Invitrogen Life Technologies, Carlsbad, CA, USA) and Polyethyleneimine MAX (Polysciences, Warrington, PA, USA), respectively, according to the manufacturer’s instructions.

### 2.2. siRNAs, in Vitro Transcribed RNA, 2-5A and Plasmids

The sequence of siRNAs for luciferase and RNase L was previously described [5]. ABCE1 siRNA consisted of 5′-r(GAU UCU AGA AGA UGA CCU A) d(TT)-3′. 5×Flag-EGFP mRNA was transcribed as described previously [5]. p5×Flag-RNase L, p5×Myc-RNase L, p5×Flag-Pelota and p5×Myc-GST were previously described [5,21]. 2-5A, a tetra-adenylate (pA2′p5′A2′p5′A2′p5′A), was synthesized, purified by reversed phase HPLC and analyzed by MALDI-TOF/MS as described previously [22]. To construct p5×Flag-ABCE1 and p5×Myc-ABCE1, cDNA encoding ABCE1 was amplified by RT-PCR using HeLa total RNA as a template and inserted into EcoRI and EcoRV sites of p5×Flag [23] and p5×Myc [24], respectively. To construct pABCE1, inverse PCR was performed to remove the Flag-tag from p5×Flag-ABCE1.

### 2.3. Northern Blotting

The decay rate of exogenous RNA was calculated as described previously [5]. HeLa cells were transfected with siRNA to downregulate the specified proteins. Forty-eight hours after siRNA-transfection, the cells were further transfected with 5×Flag-EGFP mRNA for 1 h, and then the cells were washed with phosphate-buffered saline to completely shut off supply of 5×Flag-EGFP mRNA. Subsequently, the cells were harvested at the specified time after PBS wash. Total RNA was isolated from the transfected cells using an acid guanidinium thiocyanate-phenol-chloroform extraction method and analyzed by northern blotting using DIG-labeled RNAs as described previously with minor modifications [25]. Total RNA was resolved in a 1.8% agarose/1×MOPS/2% formaldehyde gel. After overnight capillary transfer to a membrane in 20×SSC, the membrane was UV-crosslinked, prehybridized in DIG Easy Hyb (Sigma-Aldrich, St. Louis, MO, USA) at 68 °C for 1 h and then hybridized with DIG-labeled RNA probes diluted in DIG Easy Hyb (Sigma-Aldrich, St. Louis, MO, USA) at 68 °C overnight. The membrane was washed twice with 2×SSC/0.1% SDS for 5 min at r.t., twice with 0.1×SSC/0.1% SDS for 20 min and 15 min at 68 °C, and once with 1×Washing buffer (Sigma-Aldrich, St. Louis, MO, USA) for 2 min at r.t. The membrane was then blocked with 1% blocking/ 1×Maleic acid buffer (Sigma-Aldrich, St. Louis, MO, USA) for 1 h, followed by incubation in anti-DIG (1:20,000) (Sigma-Aldrich, St. Louis, MO, USA)/1% blocking/1×Maleic acid buffer for 30 min. The membrane was washed twice with 1×Washing buffer for 15 min at r.t., and then equilibrated in 1×Detection buffer (Sigma-Aldrich, St. Louis, MO, USA) for 5 min. The signals were developed using CDP-Star (Sigma-Aldrich, St. Louis, MO, USA).

### 2.4. Protein Purification

GST-RNase L protein was purified as described previously [5]. GST-RNase L was produced in *Escherichia coli* DH5α with bacterial expression vectors by adding 0.5 mM IPTG. The cells were lysed in buffer (50 mM Tris-HCl (pH 8.0), 50 mM NaCl, 1 mM DTT, 2 μg/mL aprotinin, 1 mM PMSF) and the recombinant protein was affinity-purified using Glutathione Sepharose 4B (GE Healthcare Life Sciences, Pittsburgh, PA, USA). 5×Flag-ABCE1 protein was purified from HEK293T cells. HEK293T cells expressing 5×Flag-ABCE1 were lysed in buffer (20 mM Tris–HCl (pH 7.5), 150 mM NaCl, 1 mM EDTA, 0.5% Nonidet P-40, 1 mM DTT, 10% glycerol, 0.25% sodium deoxycholate, 5 μg/mL RNase A and 1×protease inhibitor cocktail (nacalai tesque, Kyoto, Japan)). The lysate was immunoprecipitated with anti-Flag M2 agarose (Sigma-Aldrich, St. Louis, MO, USA, A2220) at 10 °C for 1 h. After five washes with wash buffer (20 mM Tris–HCl (pH 7.5), 500 mM NaCl, 0.5% Nonidet P-40, 1 mM DTT and 10% glycerol), the precipitated protein was eluted with elution buffer (20 mM Tris–HCl (pH 7.5), 500 mM NaCl, 0.5% Nonidet P-40, 1 mM DTT, 10% glycerol and 500 μg/mL FLAG peptide). The eluted protein was purified and concentrated with Amicon Ultra 30K (Merck Millipore, Darmstadt, Germany).

### 2.5. Immunoprecipitation and Western Blotting

Immunoprecipitation analyses were performed as described previously [5]. After the cells were lysed in buffer A (20 mM Tris–HCl (pH 7.5), 100 mM NaCl, 1 mM MgCl_2_, 0.5% Nonidet P-40, 1 mM DTT and 1×protease inhibitor cocktail (nacalai tesque, Kyoto, Japan)) or buffer B (20 mM Tris–HCl (pH 7.5), 100 mM NaCl, 1 mM EDTA, 0.5% Nonidet P-40, 1 mM DTT, 10% glycerol, 0.25% sodium deoxycholate, 5 μg/mL RNase A and 1×protease inhibitor cocktail (nacalai tesque, Kyoto, Japan)) on ice for 30 min, the lysate was then centrifuged at 20,400× *g* for 20 min, and the supernatant was subsequently rotated with anti-Flag M2 agarose (Sigma-Aldrich, St. Louis, MO, USA, A2220) at 10 °C for 1 h. The agarose resin was then washed three times with each buffer, and proteins retained on the agarose resin were eluted using SDS-PAGE sample buffer for western blotting.

### 2.6. Antibodies

Antibodies used for western blotting were as follows: anti-Pelota (raised against His-tagged Pelota(220-385)) [5], anti-RNase L (raised against His-tagged RNase L(1-333)) [5], anti-GAPDH (raised against His-tagged GAPDH) [26], anti-ABCE1 (raised against His-tagged ABCE1), anti-Flag (Sigma-Aldrich, MO, USA, F3165, Cell Signaling Technology, 2368, Danvers, MA, USA, 2368) and anti-Myc (Sigma-Aldrich, St. Louis, MO, USA, 11667149001, Santa Cruz Biotechnology, sc-789, Dallas, TX, USA, sc-789).

### 2.7. Statistical Analysis

*p*-values were determined using the two-tailed Student’s t test for paired samples. * *p* < 0.05, ** *p* < 0.01. Error bars represent mean ± SEM.

## 3. Results

### 3.1. ABCE1 Directly Binds RNase L

**Figure 1:**
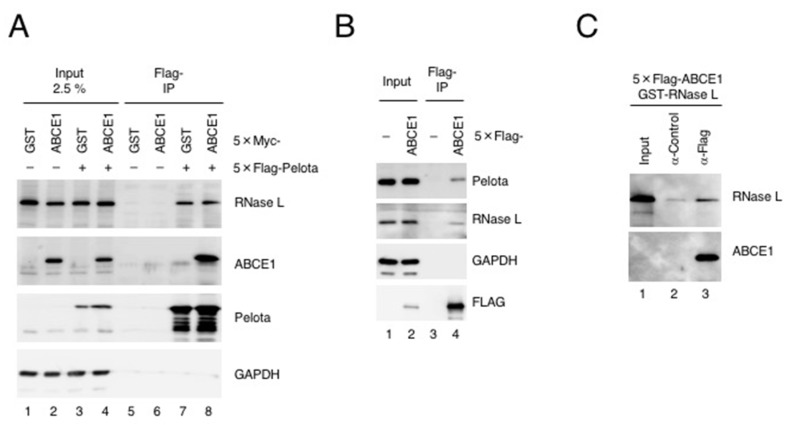
ABCE1 directly binds RNase L. (**A**) HeLa cells expressing 5×Myc-GST or 5×Myc-ABCE1 in combination with 5×Flag-Pelota were lysed in buffer B and the lysates were subjected to immunoprecipitation with anti-Flag-M2 agarose. Proteins coprecipitated with 5×Flag-Pelota were analyzed by western blotting with the indicated antibodies (*n* = 2). (**B**) HeLa cells expressing 5×Flag-ABCE1 were lysed in buffer B and the lysates were subjected to immunoprecipitation with anti-Flag-M2 agarose. Endogenous proteins coprecipitated with 5×Flag-ABCE1 were analyzed by western blotting with the indicated antibodies (*n* = 2). (**C**) Purified 5×Flag-ABCE1 and recombinant GST-RNase L were incubated with Protein G Sepharose 4 Fast Flow mixed in advance with either anti-Flag antibody or mouse IgG (control) in buffer B at 10 °C for 1 h. GST-RNase L coprecipitated with 5×Flag-ABCE1 was analyzed by western blotting with the indicated antibodies (*n* = 2).

Previous studies reported that ABCE1 interacts with eRF1 or Pelota to dissociate ribosomes either after translation termination of normal mRNAs or after recognition of stalled ribosomes on aberrant mRNAs, respectively. Interestingly, RNase L interacts with the translation factors: Pelota and eRF3 (a cofactor of eRF1) [5,20]. Moreover, we showed that RNase L directly binds to Pelota when RNase L is activated to form a dimer [5]. These findings led us to speculate that ABCE1 may form a complex with RNase L. To test this idea, we performed a coimmunoprecipitation assay using deoxycholate-solubilized cell extract, where RNase L forms a dimer in the absence of 2-5A to a level comparable to that seen in the presence of 2-5A (Supplementary Figure S1). Under this condition, we validated interactions among Pelota, ABCE1 and RNase L (Figure 1A,B). 5×Flag-Pelota coprecipitated with endogenous RNase L and 5×Myc-ABCE1 (Figure 1A) and 5×Flag-ABCE1 coprecipitated with endogenous Pelota and endogenous RNase L (Figure 1B). Notably, ABCE1 neither enhanced nor reduced the interaction between Pelota and RNase L (Figure 1A). To examine if the interaction between RNase L and ABCE1 was direct or not, we next purified GST-RNase L from *E. coli* and 5×Flag-ABCE1 from HEK293T cells and then performed a pull-down experiment. 5×Flag-ABCE1 coprecipitated with GST-RNase L (Figure 1C), indicating that ABCE1 directly binds to form a complex with RNase L. We have previously reported that RNase L also directly interacts with Pelota [5]. Furthermore, previous study reported that ABCE1 directly interacts with carboxy-terminal domain of Pelota on the ribosome [18]. Considering these findings, together with the fact that ABCE1 directly binds to RNase L, it is reasonable to assume that ABCE1, RNase L and Pelota are functionally related.

### 3.2. ABCE1 Functions in the Decay of Exogenous RNA

**Figure 2:**
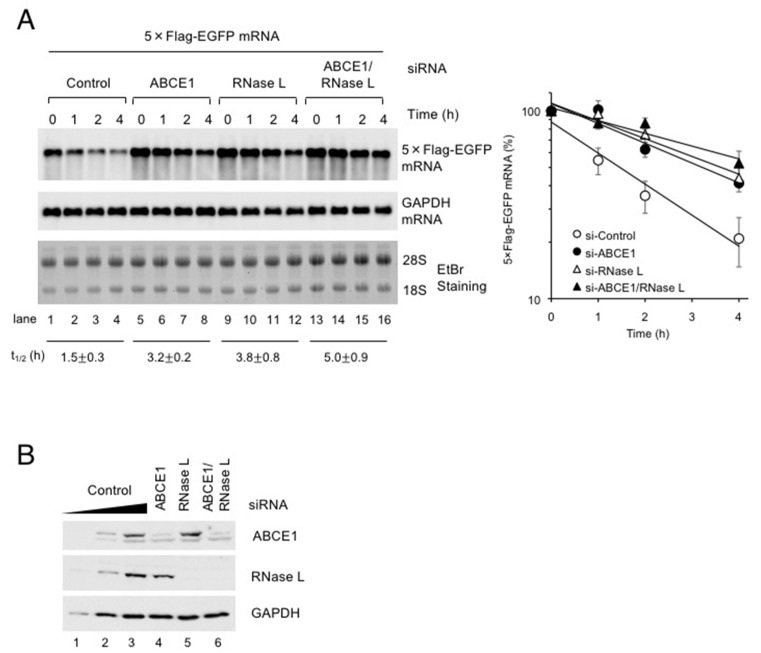
ABCE1 and RNase L act together to eliminate exogenous RNA. (**A**) ABCE1 and RNase L were depleted in HeLa cells by using siRNA. Following depletion of ABCE1 and/or RNase L, the cells were further transfected with 5×Flag-EGFP mRNA for 1 h and cultured in growth medium over time. 5×Flag-EGFP mRNA and GAPDH mRNA were analyzed by northern blotting. The leftmost five lanes analyzed two-fold dilutions of total RNA as standard curve. The levels of 5×Flag-EGFP mRNA were normalized to the levels of GAPDH mRNA to depict the decay rate (mean ± SEM, *n* = 3) and to calculate the half-lives (average t_1/2_ ± SEM, *n* = 3). (**B**) The levels of ABCE1 and RNase L in (**A**) were determined by western blotting (*n* = 3).

We previously reported that Pelota and RNase L act together to eliminate exogenous RNA in a manner dependent on translation [5]. The above results that ABCE1 directly binds Pelota and RNase L prompted us to investigate if ABCE1 functions in the decay of exogenous RNA as in the case of Pelota and RNase L. We have developed a method that measures the half-life of the exogenous RNA such as a viral RNA by using in vitro transcribed RNA [5]. HeLa cells were depleted of ABCE1 and/or RNase L for 48 h and then transfected with 5×Flag-EGFP mRNA. RNA isolated from the cells was analyzed by northern blotting (Figure 2A). Down-regulation of ABCE1 and RNase L expressions was confirmed by western blotting (Figure 2B). Consistent with our expectation, ABCE1 depletion stabilized exogenous RNA to a level comparable to that seen in RNase L depletion, and the increased half-life of the RNA by the single depletion of either protein is not significantly affected by the double depletion of both proteins, suggesting that ABCE1 functions in the decay of exogenous RNA in cooperation with Pelota and RNase L.

### 3.3. ABCE1 Does Not Affect RNase L Activity in HeLa Cells

**Figure 3:**
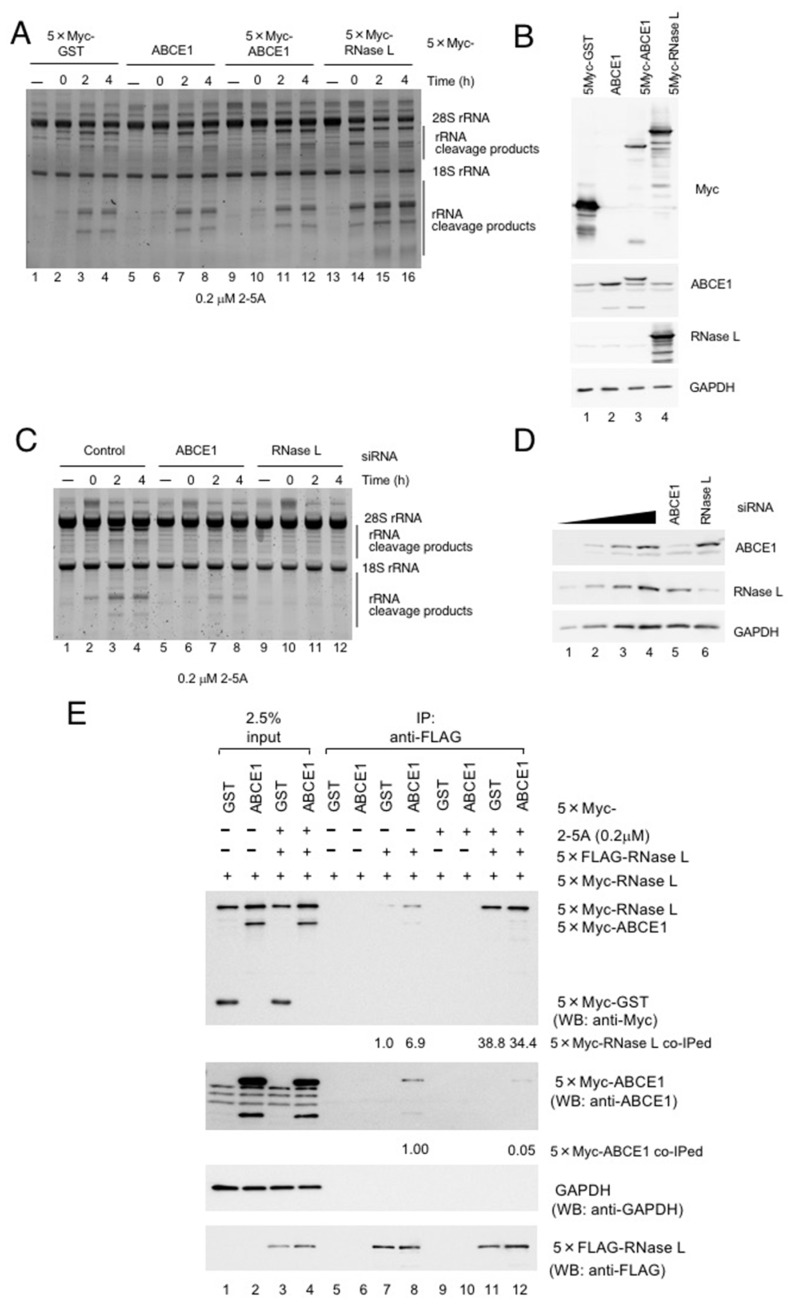
ABCE1 does not inhibit RNase L activity. (**A**) HeLa cells were transfected with p5×Myc-GST, pABCE1, p5×Myc-ABCE1 or p5×Myc-RNase L for 24 h, and the cells were further transfected with 2-5A (0.2 μM) using Neon^TM^ Transfection System. rRNAs were analyzed by SYBR-Gold staining. (**B**) The levels of proteins in (**A**) were determined by western blotting. (**C**) HeLa cells were transfected with siRNA against either luciferase (control), ABCE1 or RNase L for 48 h, and the cells were further transfected with 2-5A (0.2 μM) using Neon^TM^ Transfection System. rRNAs were analyzed by SYBR-Gold staining. (**D**) The levels of proteins in (**C**) were determined by western blotting. (**E**) HeLa cells expressing various combinations of the indicated proteins were lysed in buffer A and the lysates were rotated with anti-Flag M2 agarose in the presence or absence of 2-5A (0.2 μM). Proteins coprecipitating with 5×Flag-RNase L were analyzed by western blotting with the indicated antibodies (*n* = 2).

The above results suggest that ABCE1 is a positive regulator of exogenous RNA decay, which is inconsistent with a previous notion that ABCE1 is an inhibitor of RNase L. Thus, to obtain clues to the role of ABCE1 in exogenous RNA decay, we investigated if ABCE1 affects the enzyme activity of RNase L by detecting rRNA cleavage in HeLa cells. HeLa cells were transfected with each plasmid expressing either 5×Myc-GST (control), ABCE1, 5×Myc-ABCE1 or 5×Myc-RNase L. Twenty-four hours after plasmid-transfection, the cells were further transfected with 2-5A (0.2 μM) to activate RNase L. Activation of RNase L in HeLa cells was monitored by rRNA cleavage products. As shown in Figure 3A, rRNA cleavage products were observed two hours after 2-5A transfection (Figure 3A, lanes 1–4) and were further potentiated by RNase L transfection (Figure 3A, lanes 13–16). Overexpression of ABCE1 did not affect the degradation of rRNAs (Figure 3A, compare lanes 1–4 with lanes 5–12). The effect of ABCE1 over expression was not observed even at high concentration of 2-5A (2 μM) (Supplementary Figure S2A,B). Thus, we next examined the effect of ABCE1-depletion on the degradation of rRNA. HeLa cells were depleted of ABCE1 or RNase L for 48 h, and the cells were transfected with 2-5A (0.2 μM) to activate RNase L. As compared to the control case with luciferase siRNA, RNase L-depletion almost eliminated the rRNA cleavage products (Figure 3C, compare lanes 1–4 with lanes 9–12). In a similar manner, ABCE1 knockdown slightly but reproductively decrease the rRNA cleavage products (Figure 3C, compare lanes 1–4 with lanes 5–8). On the contrary, we could not observe the effect of ABCE1 depletion at high concentration of 2-5A (2 μM) (Supplementary Figure S2C,D). At least under our conditions, ABCE1 does not act as an inhibitor against RNase L. The levels of protein expression were confirmed by western blotting (Figure 3B,D). Next, we further investigated the ability of ABCE1 to inhibit RNase L by examining formation of active dimer of RNase L. HeLa cells were transfected with plasmids expressing two different epitope-tagged versions of RNase L, and dimer formation was assessed by coimmunoprecipitation (Figure 3E). As reported previously, Flag-tagged RNase L coimmunoprecipitated with Myc-tagged RNase L (Figure 3E, lane 7), and the interaction was markedly potentiated by the presence of 2-5A (Figure 3E, lane 11). In this condition, ABCE1 did not affect the binding between Flag-tagged RNase L and Myc-tagged RNase L (Figure 3E, lane 12). To our surprise, overexpression of ABCE1 promoted the binding between Flag-tagged RNase L and Myc-tagged RNase L (Figure 3E, compare lane 7 with lane 8). Even more interestingly, ABCE1 was released from RNase L by addition of 2-5A (Figure 3E, compare lane 8 with lane 12). Thus, it is possible that ABCE1 contributes to the dimerization of RNase L as with 2-5A and competes with 2-5A for the binding to RNase L. These results further substantiate our conclusion that ABCE1 acts as a positive regulator of exogenous RNA decay rather than an inhibitor of RNase L. 

## 4. Discussion 

**Figure 4:**
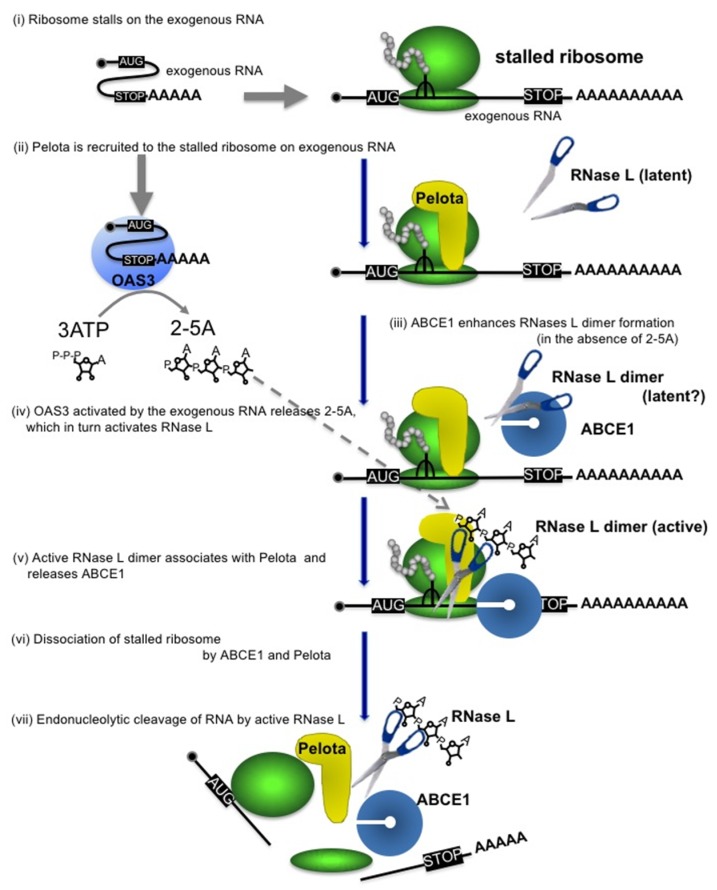
Proposed model for the ABCE1-mediated exogenous RNA decay. Pelota is recruited to the ribosome stalled on exogenous RNA. ABCE1 directly interacts with RNase L to enhance its dimerization, thereby accelerating formation of 2-5A-bound active dimer. ABCE1 released from RNase L interacts with Pelota and dissociates the ribosomes into large and small subunits. The activated RNase L degrades the exogenous RNA.

Here, we demonstrated that ABCE1 directly binds Pelota and RNase L to function as a positive regulator of exogenous RNA decay. It was unexpected that ABCE1 does not inhibit but rather promotes the activity and dimerization of RNase L. From these results together with our previous observations [5], we propose the following model for exogenous RNA decay (see also Figure 4); (i) ribosome stalls at a secondary structure formed on exogenous RNA [27], (ii) Pelota recognizes the empty A site of the stalled ribosome [5], (iii) meanwhile, ABCE1 enhances dimerization of RNase L (Figure 3E), (iv) OAS3 activated by the exogenous RNA produces 2-5A, which in turn activates RNase L [5], (v) activated RNase L dimer associates with Pelota [5] and then releases ABCE1 (Figure 3E), (vi) ABCE1 binds to form a complex with Pelota (Figure 1A,B) to dissociate the stalled ribosome, and (vii) RNase L cleaves the exogenous RNA.

ABCE1 is one of the most evolutionarily conserved proteins from archaea to mammals and has diverse functions. ABCE1 was first identified from an expression library by the ability to bind 2-5A [6]. Meanwhile, the evidence for a role in translation was reported in the interaction of ABCE1 with eukaryotic initiation factors and the 40S ribosome subunit, implicating that ABCE1 might play a role in translation initiation [9,10,28,29]. Furthermore, ABCE1 is now well studied as a ribosome recycling factor in translation termination and mRNA quality controls together with eRF1 and Pelota, respectively [14,16,17,18,19]. We recently demonstrated that Pelota/Dom34 recognizes stalled ribosomes on exogenous RNA and dissociates the ribosomes to trigger the exogenous RNA decay [5]. Since ribosome dissociation by Pelota depends on ATP-hydrolysis by ABCE1 [30], it is reasonable to assume that ABCE1 is also involved in the dissociation of stalled ribosomes on exogenous RNA (Figure 4). In this study, we have shown for the first time that ABCE1 acts as a positive regulator of exogenous RNA decay. Furthermore, we have proposed that RNase L selectively degrades exogenous RNAs with Pelota-ABCE1 in a manner dependent on translation. Most recently, two reports have shown that under conditions that cells are treated with a potent trigger poly(I:C), RNase L degrades endogenous mRNAs, which is called 2-5A-mediated decay (2-5AMD) [31,32]. Interestingly, Rath et al. demonstrated that RNase L activated by dsRNA degrades endogenous mRNAs, while RNase-L-resistant poly(A)^+^ transcripts are enriched with noncoding RNAs, suggesting that RNase L sensitivity of RNA correlates with their translational activity [31]. The 2-5AMD is similar to the exogenous RNA decay in the sense that it seems to be dependent on 2-5A, RNase L and translation; however, in our condition that measures exogenous RNA decay, we detect specifically the degradation of exogenous RNA but can neither detect degradation of endogenous mRNAs nor rRNAs (e.g., Figure 2A, see GAPDH mRNA and rRNA). In addition, we showed here that the effect of ABCE1 depletion on the 2-5A-induced rRNA cleavage was observed at lower concentration (0.2 μM) but not at higher concentration (2 μM) of 2-5A (Figure 3C and Supplementary Figure S2). Therefore, under the condition where a potent trigger poly(I:C) induces degradation of endogenous mRNA and rRNA as described in the two papers, RNase L might be fully activated and the regulation by ABCE1 might be bypassed. 

The exogenous RNA decay is thought to function as an antiviral defense mechanism that eliminates exogenous viral RNA and our previous study identified Pelota as well as RNase L as restriction factors for EMCV replication [5]. In contrast, we also identified ABCE1 as a factor promoting rather than restricting EMCV replication, which appears to be somewhat inconsistent with the notion that ABCE1 acts as a positive regulator of exogenous RNA decay. In this context, it has been reported that ABCE1 influences viral replication process [6,7,8] and functions in HIV-1 capsid assembly [11]. Although it has not been demonstrated at present whether ABCE1 is also involved in the capsid assembly of EMCV, the phenotype we have observed in the previous paper might be explained by the role in viral replication.

Although we have demonstrated ABCE1 as a positive regulator of exogenous RNA decay, it is still incompletely understood how ABCE1 enhances dimer formation of RNase L. The data in Figure 3E shows that ABCE1 enhances dimer formation in the absence of 2-5A. However, the addition of 2-5A further accelerates dimer formation and releases ABCE1 from the RNase L dimer. The fact that RNase L could form a dimer in the absence of 2-5A is novel and unexpected, but the RNase L’s potential to form a dimer in the absence of 2-5A was also observed in Figure 1 and Supplementary Figure S1. Therefore, these data suggest that ABCE1 binds RNase L in the absence of 2-5A and enhances dimer formation of RNase L. Although it is not clear at present whether the RNase L dimer formed in the absence of 2-5A is latent or active, it is reasonable to assume that ABCE1-mediated formation of RNase L dimer enhances formation of 2-5A-bound active RNase L dimer. Moreover, it is also a matter of debate whether ABCE1 dissociates stalled ribosomes on exogenous RNA to accelerate exogenous RNA decay. Further studies will be required to elucidate the precise roles of ABCE1 on exogenous RNA decay.

## Supplementary Materials

The following are available online at https://www.mdpi.com/1999-4915/12/2/174/s1.
