# Supplementary material for: ABCE1 Acts as a Positive Regulator of Exogenous RNA Decay
Source: Viruses. 2020 Feb 4;12(2):174. doi: 10.3390/v12020174 (PMC7077301; doi:10.3390/v12020174)
Supplement: Supplementary file 1 [file viruses-12-00174-s001.pdf]

Figure S1

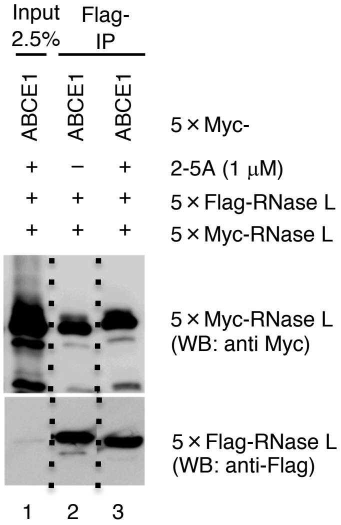

Figure S1. RNase L forms a dimer in buffer containing sodium deoxycholate.
HeLa cells expressing various combinations of the indicated proteins were lysed in buffer B and the lysates were rotated with anti-Flag M2 agarose in the presence or absence of 2-5A (1 μM). Proteins co-precipitating with 5 × Flag-RNase L were analyzed by western blotting with the indicated antibodies. Flag-tagged RNase L co-immunoprecipitated with Myc-tagged RNase L in the presence of 2-5A (lane 3) as reported previously, while under this assay condition, even in the absence of 2-5A we could detect co-precipitation of RNase L (lane 2) to a level comparable to that seen in the presence of 2-5A (compare lane 2 and lane 3).

Figure S2

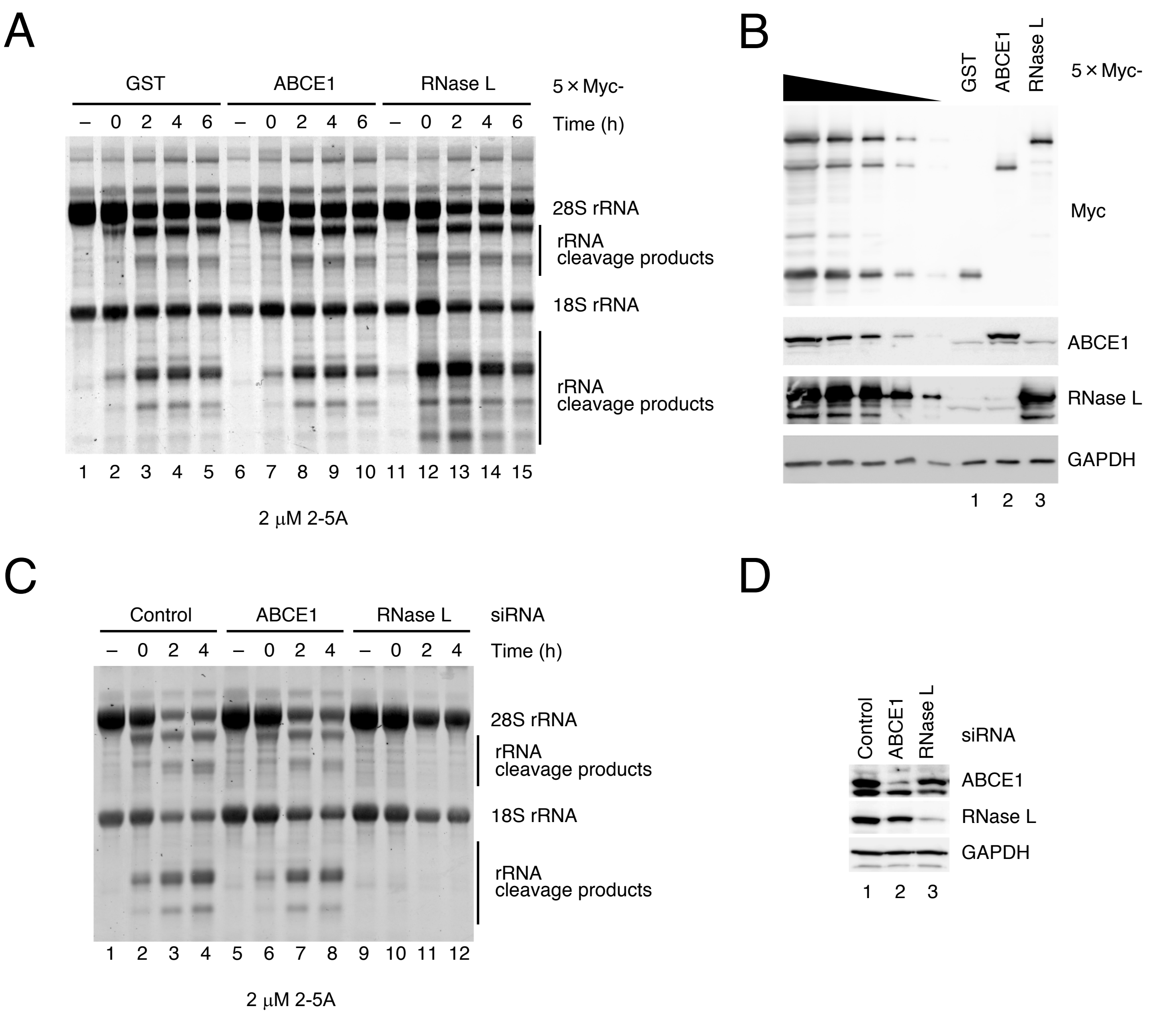

Figure S2. ABCE1 does not affect RNase L activity related to Figure 3. (A) HeLa cells were transfected with p5 × Myc-GST, pABCE1, p5 × Myc-ABCE1 or p5 × Myc-RNase L for 24 hours, and the cells were further transfected with 2-5A (2 mM) using Neon™ Transfection System. rRNAs were analyzed by SYBR-Gold staining. (B) The levels of proteins in (A) were determined by western blotting. (C) HeLa cells were transfected with siRNA against either luciferase (control), ABCE1 or RNase L for 48 hours, and the cells were further transfected with 2-5A (2 mM) using Neon™ Transfection System. rRNAs were analyzed by SYBR-Gold staining. (D) The levels of proteins in (C) were determined by western blotting.
